# Eupalinolide B inhibits periodontitis development by targeting ubiquitin conjugating enzyme UBE2D3

**DOI:** 10.1002/mco2.70034

**Published:** 2025-01-14

**Authors:** Wenhua Kuang, Ruishen Zhuge, Ping Song, Letai Yi, Shujie Zhang, Ying Zhang, Yin Kwan Wong, Ruixing Chen, Junzhe Zhang, Yuanbo Wang, Dandan Liu, Zipeng Gong, Peili Wang, Xiangying Ouyang, Jigang Wang

**Affiliations:** ^1^ Department of Urology, Guangdong Provincial Clinical Research Center for Geriatrics, Shenzhen Clinical Research Centre for Geriatrics Shenzhen People's Hospital, The First Affiliated Hospital, Southern University of Science and Technology Shenzhen China; ^2^ Department of Periodontology, National Clinical Research Center for Oral Diseases, National Engineering Laboratory for Digital and Material Technology of Stomatology, Beijing Key Laboratory of Digital Stomatology Peking University School and Hospital of Stomatology Beijing China; ^3^ National Clinical Research Center for Chinese Medicine Cardiology Xiyuan Hospital, China Academy of Chinese Medical Sciences Beijing China; ^4^ Inner Mongolia Medical University Hohhot China; ^5^ State Key Laboratory for Quality Ensurance and Sustainable Use of Dao‐di Herbs, Artemisinin Research Center, Institute of Chinese Materia Medica China Academy of Chinese Medical Sciences Beijing China; ^6^ State Key Laboratory of Functions and Applications of Medicinal Plants, Guizhou Provincial Key Laboratory of Pharmaceutics Guizhou Medical University Guiyang China; ^7^ State Key Laboratory of Antiviral Drugs, School of Pharmacy Henan University Kaifeng China

**Keywords:** chemical biology, drug targets, natural product, proteomics, target identification

## Abstract

Periodontitis is a chronic periodontal inflammatory disease caused by periodontal pathogens commonly seen in adults. Eupalinolide B (EB) is a sesquiterpenoid natural product extracted from Eupatorium lindleyanum and has been reported as a potential drug for cancers and immune disorders. Here, we explored the ameliorative effects and underlying molecular mechanism of EB on periodontitis for the first time. We demonstrated that EB ameliorates periodontal inflammation and alveolar bone resorption with a ligated periodontitis mouse model. In addition, the impact of EB on macrophages inflammation was examined in the Raw264.7 cell line. We identified ubiquitin‐conjugating enzyme, UBE2D3, as the direct covalent binding protein targets of EB by using a chemoproteomic method based on activity‐based protein profiling, biolayer interferometry method, and cellular thermal shift assay. Furthermore, the direct binding site of EB to UBE2D3 was identified using high‐resolution mass spectrometry and confirmed by experiments. Taken together, EB ameliorates periodontitis by targeting UBE2D3 to suppress the ubiquitination degradation of IκBα, leading to inactivation of nuclear transcription factor‐κB signaling pathway. And this was confirmed by siRNA‐mediated gene knockdown in inflammatory macrophages. Our results suggested that EB may be a new kind of UBE2D3 inhibitor and may become a promising therapeutic agent for anti‐periodontitis.

## Graphical abstract

This study shows application of a small molecule compound Eupalinolide B in periodontitis, which effectively alleviates periodontal inflammation and alveolar bone loss. By using an array of chemical biology approaches, direct binding protein target, UBE2D3, and mechanisms of actions of Eupalinolide B in the treatment of periodontitis were revealed.

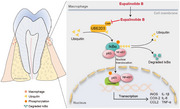

## 1 INTRODUCTION

Periodontitis, a frequently encountered oral disease, is characterized by continuous gingival inflammation, alveolar bone resorption, and eventually tooth loss. Epidemiological investigation showed that more than 50% adults suffered from periodontitis worldwide, and about 8% severe periodontitis.^1^ Periodontitis can also lead to some chronic inflammation‐driven systemic diseases, causing initiation or aggravation of neurodegenerative disorders, autoimmune diseases, cardio‐metabolic diseases, and cancer.^2^, ^3^, ^4^, ^5^ In view of the severity and risk of periodontitis, to study the prevention and treatment of periodontitis is of great significance. Drugs targeting to inhibit inflammatory response and alveolar bone resorption have become the emphasis and hotspots of research on prevention and treatment for periodontitis. Clinically, conventional periodontal mechanical therapy associated with pharmacotherapy, including anti‐bacterials, anti‐osteoporosis drugs, and non‐steroidal anti‐inflammatory drugs, could improve the curative effects of periodontitis treatment.^6^, ^7^, ^8^, ^9^ However, the therapeutic effect of medicine on periodontitis is not satisfactory due to potential adverse reactions and persistent innate immune responses.^10^ Therefore, it is very necessary to explore an effective drug for treating periodontitis with immunomodulation role and insignificant side effects.

Macrophages, as inherent immune cells in periodontal tissues, are essential for maintaining microenvironment homeostasis and participate in resisting periodontal pathogens, preventing periodontal tissue damage.^11^, ^12^, ^13^, ^14^ During periodontitis, macrophages undergo corresponding phenotypic transformation to affect disease progression.^13^, ^15^, ^16^ In periodontitis, dental plaque was recognized as the major factor leading to periodontitis.^17^ Microorganisms and their products in the periodontium can promote the activation of macrophages, and activated macrophages can secrete a variety of pro‐inflammatory factors, such as tumour necrosis factor‐alpha (TNF‐α), interleukin‐6 (IL‐6), interleukin‐1β (IL‐1β), chemokine (C‐C motif) ligand 2 (CCL‐2), cyclooxygenase‐2 (COX‐2), nitric oxide (NO), etc., which promote the occurrence and development of periodontitis, periodontal tissue damage, and alveolar bone loss.^18^ Therefore, modulating the macrophage immune response could be an alternative treatment approach for periodontitis.

Nuclear transcription factor‐κB (NF‐κB) pathway is highly activated in the macrophages of tissues from patients with periodontitis than healthy gingival tissue.^19^ During periodontitis, *Porphyromonas gingivalis*‐lipopolysaccharide (Pg‐LPS) is regarded as one of the main pathogenic factor.^20^ Pg‐LPS promotes the production of inflammatory factors mainly by activating NF‐κB pathway.^21^ As a classical pro‐inflammatory signaling, NF‐κB binds to IκB, the inhibitory protein, in the cytoplasm under normal conditions. IκB kinase (IKK) is activated when cells are stimulated by Pg‐LPS. Then, IκBα is phosphorylated and degraded through the ubiquitin‐proteasome‐dependent pathway, resulting in nuclear translocation of NF‐κB members such as the p50/c‐Rel or p50/p65 dimers.^22^, ^23^ The progression of periodontal inflammation was mediated by the upregulated pro‐inflammatory gene transcripts in innate immune response triggered by the NF‐κB signaling pathway.^24^, ^25^, ^26^, ^27^ Inhibiting the activation of NF‐κB p65 subunits and increasing the expression of IκBα and therefore inhibiting NF‐κB pathway could improve periodontitis.^28^, ^29^

UBE2D3 is an important ubiquitin‐conjugating enzyme (E2) which can cooperate with ubiquitin ligase (E3) to transfer ubiquitins to IκBα, the inhibitory protein of NF‐κB. Then, IκBα is ubiquitinated and further degraded so that promote NF‐κB activation and nuclear translocation.^30^, ^31^ In addition to directly promoting IkBα ubiquitination, the Ube2d3 can also activate IKK to inhibit IkBα.^32^, ^33^ This suggests that it is a potential strategy to develop a novel NF‐κB inhibitor that targeting Ube2d3.^31^, ^34^ Ube2d3 is abnormally highly expressed in a variety of human cancers and immune diseases and participated in the progression of these diseases such as esophageal, breast, pancreatic, and colorectal cancer.^35^, ^36^, ^37^ In recent years, some Ube2d3 inhibitors have been developed to inhibit the activity of Ube2d3 by forming covalent adjuncts,^38^ forming an active area of drug development. Some studies have shown that UBE2D3 inhibitors could reduce inflammatory response in colitis and lung injury models based on target protein prediction method.^39^ Targeting UBE2D3 is therefore a potential strategy for developing anti‐inflammatory diseases drugs.

Eupalinolide B (EB), is one of the major sesquiterpenoids extracted from Eupatorium lindleyanum DC.^40^ Previous studies during last years have shown that EB exhibited roles of anti‐neuritis, anti‐acute lung injury, and anti‐hepatic carcinoma.^41^, ^42^, ^43^ However, the effects of EB on periodontitis remain unclear and its direct target and mechanism still need to be further evaluated. In this study, we examined the therapeutic role of EB on periodontitis, synthesized the specific probe of EB and screened the direct target protein UBE2D3 by activity‐based protein profiling (ABPP) method using this probe for the first time. We clarified the underlying molecular mechanism by which EB inhibits the NF‐κB inflammatory signaling pathway based on proteomics of mouse periodontal tissue. In fact, it is more rational to obtain the target and underlying pathway regulated by EB through high‐throughput screening technology. Also, we identified the covalent binding of EB to the Cys85 residue of UBE2D3 protein by high‐resolution mass spectrometry and further proved that Cys85 is critical for the combination of EB and UBE2D3 protein identified by ABPP. Inhibition of UBE2D3‒NF‐κB signaling by EB may contribute to alleviating the progression of periodontitis.

## 2 RESULTS

### 2.1 EB exerts anti‐periodontal inflammation as well as alveolar bone loss in vivo

**FIGURE 1:**
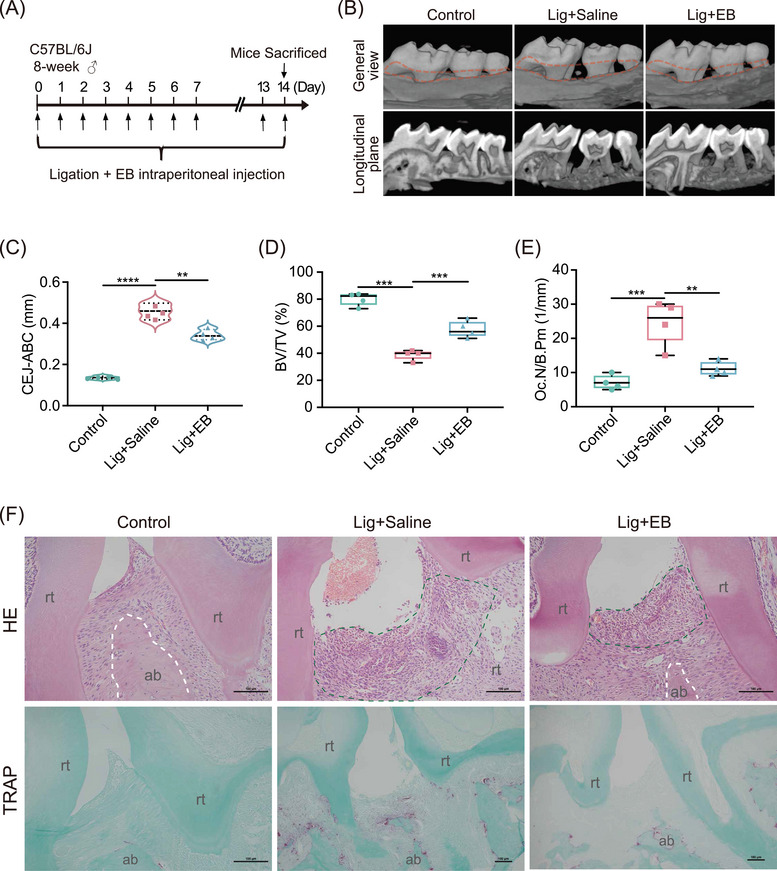
Eupalinolide B alleviates periodontitis in vivo. (A) The flow chart of the experiment in ligated C57BL/6 mice. (B) Three‐dimensional reconstruction of micro‐computed tomography (micro‐CT) scanning of the alveolar bone in mice (*n* = 4). (C) Cemento‐enamel junction and alveolar bone crest (CEJ‒ABC) distance (*n* = 4). (D) Bone volume/total volume (BV/TV) (*n* = 4). (E) Number of osteoclasts/per bone circumference (*n* = 4). (F) Representative figures of hematoxylin‒eosin (upper) and tartrate‐resistant acid phosphatase (lower) staining of the periodontal tissue. Lig, ligature. Scale bar: 100 µm. Data are represented as means ± SEM. ^*^ *p* < 0.05, ^**^ *p* < 0.01, and ^***^ *p* < 0.001.

To explore whether EB was protective against periodontitis, an experimental model of ligature‐induced periodontitis based on C57BL/6 mice was established. Mice were intraperitoneally injected with or without EB daily for 14 days to evaluate the effects on periodontitis‐induced inflammation and alveolar bone resorption (Figure 1A). The distance between the cemento‐enamel junction and alveolar bone crest (CEJ‒ABC) is a clinical index to evaluate the alveolar bone resorption (Figure 1B,C). Bone volume/tissue volume (BV/TV) percentage was used to quantify bone mineral density and explain changes in bone mass. And the periodontal maxillary resorption was significant in the Lig + saline group, while alveolar bone resorption was alleviated in the Lig + EB group compared with the control group (Figure 1B‒D). Hematoxylin and eosin (HE) staining demonstrated that EB can alleviate inflammatory cell infiltration and relieve alveolar bone destruction in experimental periodontitis mice (Figure 1F). Tartrate‐resistant acid phosphatase (TRAP) staining results showed that osteoclasts increased in the Lig + saline group compared with the control group. Notably, EB treatment actually reduced the osteoclasts in alveolar bone in mice model with ligated periodontitis (Figure 1E,F), suggesting that bone resorption was significantly higher in the Lig + saline group as compared to the control group while EB attenuated the undesirable trend of alveolar bone resorption. These results indicate that EB can effectively improve periodontal inflammatory injury and inhibit periodontal destruction.

### 2.2 Proteomic analysis proved a participation of the NF‐κB signaling pathway in anti‐periodontitis effect of EB

**FIGURE 2:**
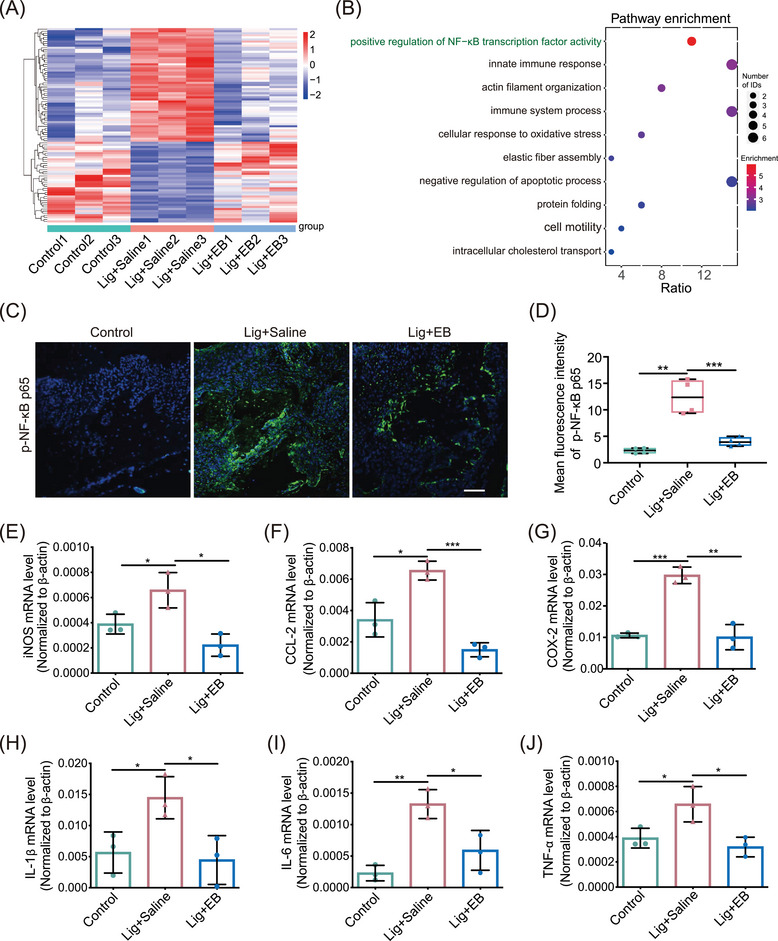
Proteome in mouse gingival tissues identifies downregulated pathways. (A) Heatmap of proteomes in ligated periodontal groups with or without Eupalinolide B (EB) treatment and normal control groups (*n* = 3). (B) Gene ontology analysis for the significantly downregulated pathways by EB in gingival tissues compared to ligated group. The top 10 regulated pathways were demonstrated. (C and D) Activated nuclear transcription factor‐κB (NF‐κB) p65 was explored by immunofluorescence in alveolar bone tissues (*n* = 3). Scale bar: 50 µm. (E‒J) The relative mRNA levels of genes indicated in mice gingival tissues were measured using qRT‐PCR (*n* = 3). Data are represented as means ± SEM. ^*^ *p* < 0.05, ^**^ *p* < 0.01, or ^***^ *p* < 0.001.

To further investigate the underlying mechanism by which EB inhibits periodontitis, we performed an overall proteomic analysis of mouse gingival tissues. Principal component analysis and heatmap showed that there were significant differences between the control, Lig + saline, and Lig + EB groups (Figures 2A and S1A). Compared with the periodontitis group, 112 proteins were significantly decreased (fold change < 0.8 and *p*‐value < 0.05) and 362 proteins increased (fold change > 1.2 and *p*‐value < 0.05) in EB‐treated group (Table S1). Then, Gene Ontology and Kyoto Encyclopedia of Genes and Genomes (KEGG) pathway analyses were applied to analyze the differentially expressed proteins (Figures 2B and S1B‒D). Notably, EB administration downregulated the “positive regulation of NF‐κB transcription factor activity,” “immune system processes,” and “innate immune response” pathway (Figure 2B). It has been well known that as a transcription factor, NF‐κB plays an crucial role in the inflammatory cytokine storm induced by Pg‐LPS in periodontitis. When stimulated by Pg‐LPS, NF‐κB is continuously activated in cells through a series of signal transduction and phosphorylated NF‐κB p65 (p‐NF‐κB p65, activated form) driving the nucleus translocation of p65, eventually leading to the transcription and expression of downstream target genes (*IL‐1β*, *IL‐6*, *TNF‐α*, *iNOS*, *COX‐2*, *CCL‐2*, etc.) related to immune and inflammatory responses.^44^, ^45^ The innate immune response triggered by the NF‐κB pathway contributes to the progression of periodontitis. In order to investigate the inhibition of NF‐κB activation by EB in vivo, the distribution and expression of p‐NF‐κB p65 in mouse gingival tissues were detected by immunofluorescence staining. As displayed in Figure 2C, p‐NF‐κB p65 fluorescence was significantly enhanced in the gingival tissue of the second molar periodontal region of mice in Lig + saline group compared with the control group, while EB administration inhibited p‐NF‐κB p65 expression (Figure 2C,D). Furthermore, we analyzed the transcription levels of downstream genes of NF‐κB in the adjacent gingival tissue of the ligation site. The results showed that the mRNA levels of *iNOS*, *CCL‐2*, *COX‐2*, *IL‐1β*, *IL‐6*, and *TNF‐α* in Lig + saline group were significantly higher than those in the control group while EB significantly reduced the transcription levels (Figure 2E‒J). These results indicate that EB can inhibit experimental periodontitis by inhibiting the NF‐κB transcription factor activity in vivo.

### 2.3 EB inhibits the NF‐κB pathway induced by Pg‐LPS

**FIGURE 3:**
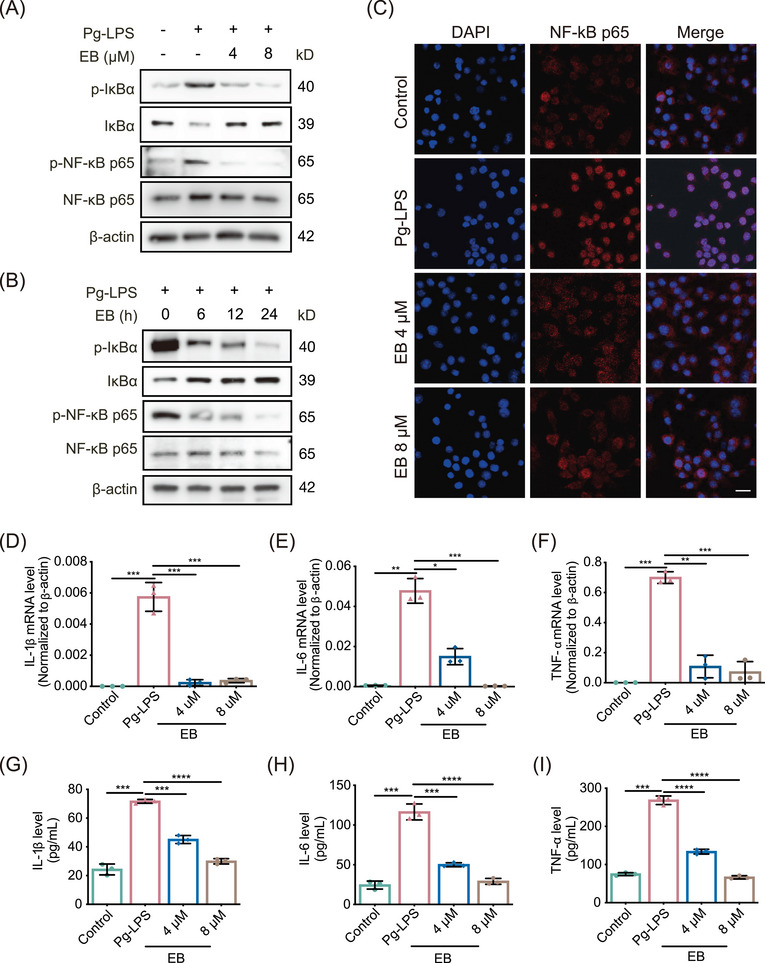
Eupalinolide B (EB) inhibits nuclear transcription factor‐κB (NF‐κB) signaling pathway in Raw264.7 cells. (A) The protein levels of phospho‐IκBα, IκBα, phospho‐NF‐κB p65, and NF‐κB p65 were measured using western blot under EB treatment after stimulated with *Porphyromonas gingivalis*‐lipopolysaccharide (Pg‐LPS) for 24 h (*n* = 3). (B) The indicated proteins phospho‐IκBα, IκBα, phospho‐NF‐κB p65, and NF‐κB p65 were measured by western blot after EB (8 µM) treatment (*n* = 3). (C) The distribution of NF‐κB p65 was detected by immunofluorescence staining in Raw264.7 cells (*n* = 3). Scale bar: 100 µm. (D‒F) The relative mRNA levels of indicated genes (interleukin‐6 [IL‐6], tumor necrosis factor‐alpha [TNF‐α], and IL‐1β) downstream of NF‐κB pathway were determined by qPCR (*n* = 3). (G‒I) Cytokines (TNF‐α, IL‐6, and IL‐1β) of target gene products downstream of NF‐κB pathway released in the supernatant of cell culture were detected by enzyme‐linked immunosorbent assay (*n* = 3). Data are represented as means ± SEM. ^*^ *p* < 0.05, ^**^ *p* < 0.01, ^***^ *p* < 0.001, or ^****^ *p* < 0.0001.

The effects of EB on activation of NF‐κB signaling pathway induced by Pg‐LPS was further assessed. We first evaluated the cytotoxicity of EB probe (EB‐P) using cell counting kit‐8 (CCK‐8) assay and found that EB‐P displayed non‐cytotoxicity in RAW264.7 cells even up to 16 µM (Figure S2A). And we found that the phosphorylation of IκBα was significantly inhibited by EB both at the lower concentrations of 4 and 8 µM (Figure 3A,B). Besides, IκBα was significantly stabilized and the phosphorylated NF‐κB p65 levels was downregulated by EB upon Pg‐LPS treatment in Raw264.7 cells (Figure 3A,B). We also found that, after Pg‐LPS stimulation, EB significantly inhibited nuclear translocation of NF‐κB p65 (Figure 3C). To further elucidate the consequences of reduced NF‐κB‐driven transcriptional activity of EB‐treated Raw264.7 cells, qPCR was conducted to measure the transcript levels of *TNF‐α*, *IL‐6*, and *IL‐1β*, downstream genes of NF‐κB pathway (Figure 3D‒F). In addition, the secretion of related inflammatory factors above were also measured using enzyme‐linked immunosorbent assay (ELISA) (Figure 3G‒I). Results demonstrated that the mRNA levels and secretion levels of inflammatory cytokines above were all significantly inhibited compared with the control group in EB‐treated cells.

### 2.4 ABPP proteomic analysis confirmed UBE2D3 as target protein of EB

**FIGURE 4:**
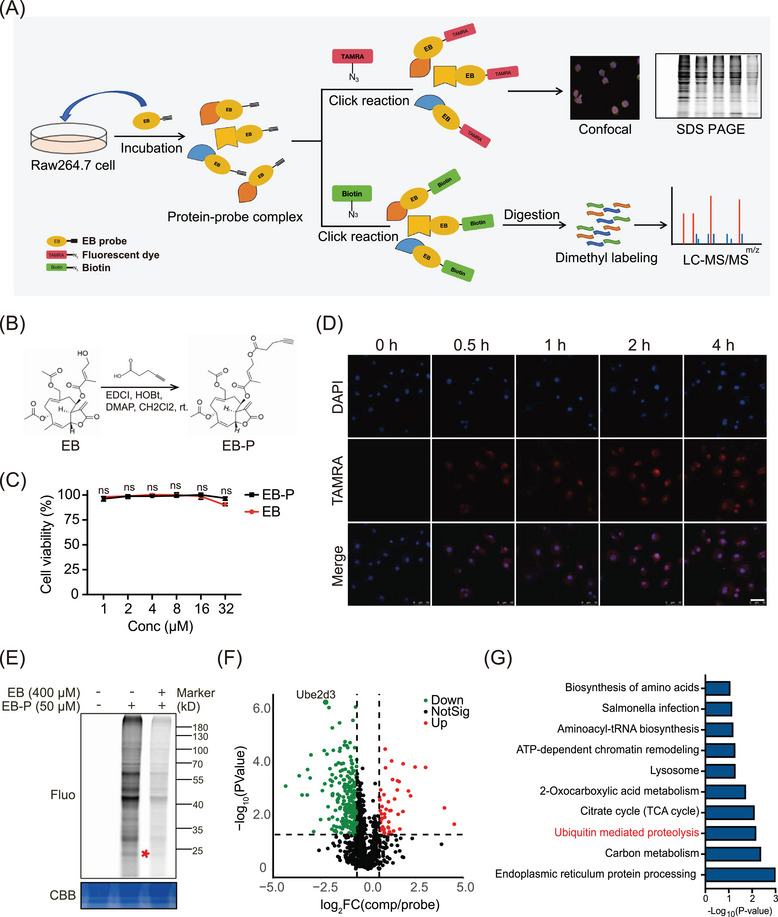
Identification of target proteins of Eupalinolide B (EB) in Raw264.7 cells. (A) Overall flow chart of target protein identification by activity‐based protein profiling (ABPP) analysis. (B) Chemical structures of EB and EB probe (EB‐P). (C) Cell viability of Raw264.7 cells treated with EB or EB‐P (*n* = 5). (D) Cellular imaging of EB‐P with different exposure times in RAW264.7 cells. Scale bar: 50 µm. (E) The labeling of target proteins with 50 µM EB‐P and competition by EB (400 µM) in situ. The red star represent the 17 kDa protein band. (F) Volcano plot of proteins identified in ABPP method. The graph displayed with the log_2_ (fold change [FC]) of competition group (400 µM EB + 50 µM EB‐P) versus EB‐P (50 µM) (*x*‐axis) against the ‒log_10_ *p*‐value (*y*‐axis). The dots with *p*‐value <0.05 and FC <0.6 (green) were selected as target protein candidates. (G) Kyoto Encyclopedia of Genes and Genomes (KEGG) pathway analysis of the target protein candidates.

In view of the significant anti‐inflammatory effects of EB, we further explored the target proteins and molecular mechanism of EB based on ABPP analytical technique (Figure 4A). Briefly, an activity‐based EB‐P with a clickable alkyne tag to EB was synthesized (Figure 4B). A fluorescent dye TAMRA or biotin can be linked to EB‐P via click chemical reaction to label the directly bond protein targets of EB. Then, target proteins can be identified by mass spectrometry or visualized by fluorescence imaging (Figure 4A). There were no significant differences anti‐inflammatory bioactivity between EB and EB‐P (Figure S2B,C), suggesting that EB‐P can be used to perform target identification and fluorescence imaging experiments instead of EB. We further incubated Raw264.7 cells with EB‐P for different times and conducted subcellular localization experiments. The results showed that EB‐P gradually entered cells over time and the fluorescence intensity reached the strongest after 2 h later (Figure 4D). We observed that EB‐P can label target proteins localized to cytoplasm and membranes (Figure 4D). To further identify the direct target proteins of EB via ABPP method, activated RAW264.7 cells were pre‐treated with or without EB and then labeled with 50 µM EB‐P for 4 h, the target protein EB‐P covalently labeled were coupled with fluorescent dye by click chemical reaction and then electrophoretically separated by sodium dodecyl sulfate polyacrylamide gel electrophoresis (SDS‐PAGE) and visualized through fluorescence imaging (Figure 4E). As expected, 50 µM EB‐P can label most cellular proteins and the labeling of proteins were competed off by 400 µM EB pre‐incubated in the RAW264.7 cells (Figure 4E), suggesting a specificity of EB‐P labeling proteins. Then, the EB‐P‐labeled target proteins were enriched by streptavidin agarose and digested into peptides by trypsin. Finally, the peptides were quantified by dimethylation labeling and identified by mass spectrometry. Seventy‐nine target protein candidates were screened (fold change of probe/con > 1 and competition/probe < 1, and *p*‐value < 0.05) from total 1247 proteins identified (Figure 4F) and it is worth noting that, UBE2D3, with the highest probe‐to‐competition ratio, was significantly higher in the probe group than the competition group (Figure 4F). As an ubiquitin conjugating enzyme, UBE2D3 was involved in post‐translational ubiquitination of proteins. Furthermore, KEGG pathway analysis of target protein candidates revealed that target proteins were mainly enriched in “ubiquitin‐mediated proteolysis” (Figure 4G). Consistently, we noticed that there was one of the main distinct bands observed in the EB‐P lane in SDS‐PAGE sharing the similar molecular weight with UBE2D3 protein at about 17 kDa, which was not observed in the lane without EB‐P incubation and significantly reduced in intensity in the EB competition lane (Figure 4E). Taken together, the results above indicated UBE2D3 as a potential direct target protein of EB.

### 2.5 EB directly targets UBE2D3 in RAW264.7 cells

**FIGURE 5:**
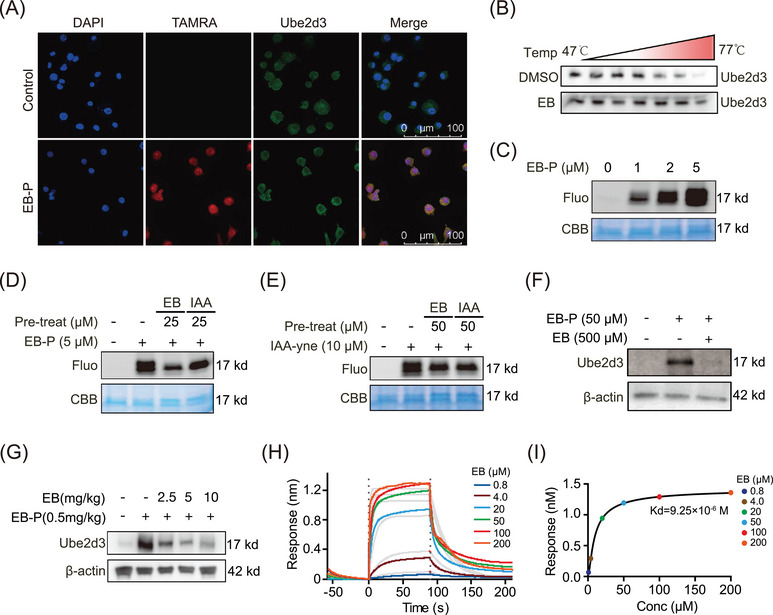
The direct interactions between Eupalinolide B (EB) and UBE2D3. (A) Co‐localization of EB probe (EB‐P) (red) and UBE2D3 (green) by immunofluorescence staining using UBE2D3 antibody. (B) The EB‐UBE2D3 binding was confirmed using cellular thermal shift assay‐western blot (CETSA‐WB) in Raw264.7 cells. (C) The EB‐P labeling of UBE2D3. (D) The competition of labeling of UBE2D3 with EB‐P with or without EB or iodoacetamide (IAA). (E) The competition of labeling of UBE2D3 with IAA‐yne with or without EB or IAA. (F) Pull‐down WB of UBE2D3 using EB‐P with or without EB in Raw264.7 cells. (G) Pull‐down WB of UBE2D3 using EB‐P with or without EB in mice gingival tissues. (H and I) The binding affinity of EB on UBE2D3 was measured by biolayer interferometry.

We investigated the localization of UBE2D3 (green) by immunofluorescence staining with antibodies upon EB‐P pre‐incubation in RAW264.7 cells, then EB‐P were linked with a TAMRA tag (red) by click reaction (Figure 5A). The results exhibited co‐localization (yellow) of UBE2D3 and EB‐P mainly in the cytoplasm (Figure 5A). Furthermore, the thermal stability of the UBE2D3 protein was evaluated by cellular thermal shift assay‐western blot (CETSA‐WB) experiment and result showed that compared with control group, EB enhanced the thermal stabilization of UBE2D3 (Figure 5B). In order to gain insight into the molecular mechanism that EB binding to UBE2D3, recombinant mouse UBE2D3 protein was treated with EB‐P and then subjected to a click chemical reaction. The results showed that recombinant UBE2D3 protein was concentration‐dependently labeled by EB‐P (Figure 5C). Next, we performed a EB‐P competition experiment on recombinant UBE2D3 protein. The UBE2D3 were pre‐treated with different concentrations of EB or iodoacetamide (IAA, a cysteine‐alkylating agent), and then incubated with EB‐P. The results showed that, similarly to EB, IAA also significantly weakened the binding of EB‐P to UBE2D3 protein, indicating that EB‐P can bind to cysteine residues of UBE2D3 (Figure 5D). Moreover, we used IAA‐alkyne based probe containing alkynyl (IAA‐yne) that can bind to cysteine via a click chemical reaction to perform a competition experiment instead of EB‐P.^46^ Notably, UBE2D3 can be labeled by IAA‐yne, while both EB and IAA can significantly compete for IAA‐yne to bind to UBE2D3 protein (Figure 5E). In addition, EB‐P could not only pull down UBE2D3 in cell lysate of activated Raw264.7 cells, and this can be competed away by EB (Figure 5F), but also effectively bond to UBE2D3 protein in gingival tissues of mice and was concentration‐dependently competed off by EB administration (Figure 5G), indicating that EB can directly bind UBE2D3 protein both in vitro and in vivo. To further confirm the direct interaction between UBE2D3 and EB and identify the dynamic properties, we used the biolayer interferometry (BLI) technique to measure the equilibrium dissociation constant between EB and UBE2D3 and found that EB significantly bound to UBE2D3 with the binding affinity (*K*_d_ value) as 9.25 µM (Figure 5H,I).

### 2.6 EB covalently targets UBE2D3 protein and binds to the Cys85 residue

**FIGURE 6:**
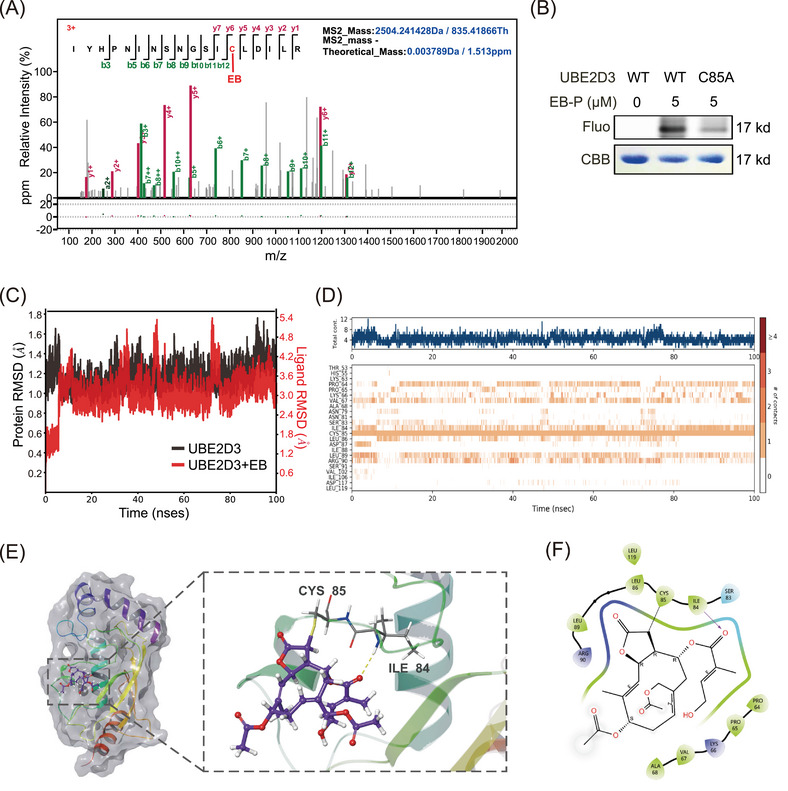
Eupalinolide B (EB) covalently bind to the cysteine residue of UBE2D3. (A) Mass spectrometry (MS)/MS spectra of precursor ions for accurate sequencing and localization of EB binding site. (B) The labeling of UBE2D3 and its mutant UBE2D3‐C85A with EB probe (EB‐P). The results are from three different experiments. (C) Root‐mean‐square deviation (RMSD) plots throughout the 100 ns molecular dynamic simulation. (D) Contact counts of the UBE2D3/EB complex. (E) Surface representation of the binding pockets of UBE2D3/EB complex generated by molecular docking. (F) Ligand interaction diagrams of the UBE2D3/EB complex.

Moreover, to determine the specific binding sites of EB to UBE2D3, the recombinant UBE2D3 was incubated with or without EB and the proteins were then digested into peptides with trypsin. The peptides were analyzed by liquid chromatography coupled with mass spectrometry‐based methods. A peptide with a molecular mass of 2504.24 with definite mass change derived from digestion of UBE2D3 protein in EB incubated samples was identified by high‐resolution mass spectrometry with high confidence (Figure 6A and Tables S2 and S3). This peptide fragment contains the active cystine Cys85 of UBE2D3, indicating that UBE2D3 was modified by EB in Cys85. Interestingly, no peptides containing other free cysteines were found to be modified by EB, indicating that there was a covalent binding of EB to UBE2D3 spatially selective for Cys85 residue. Following that, EB‐P labeling and EB binding affinity experiments were reversely validated using the UBE2D3‐C85A mutant (Figure 6B). To further derive the dynamic behavior of the residues at the active sites of UBE2D3, we further used Maestro software to conduct molecular dynamics simulations for 100 ns under the conditions of a real solution environment, and their molecular dynamics trajectories were analyzed. The conformational stability of the system was tested using the curve of root‐mean‐square deviation (RMSD) against the simulated time. Results demonstrated that the RMSD values of the α‐carbon (Cα) atoms in UBE2D3 (black) or EB‐UBE2D3 complex (red) eventually reached an equilibrium state with small fluctuations after 10 ns (Figure 6C), indicating that the complex structures reached stable states during the simulations. The interaction fraction was also monitored for the EB‐UBE2D3 complex, EB formed hydrogen bonds directly with ILE84 (40%), LEU86 (23%), and LEU89 (14%), hydrogen bonds indirectly with LEU89 (15%), PRO64 (32%), and VAL67 (35%) via water bridges, as well as hydrophobic interactions with CYS85 (99%) and LEU89 (10%) (Figure S3A,B). There were continuous contacts generated by EB with the amino acids ILE84, CYS85, and LEU89 (contacts are shown in orange) across the whole molecular trajectories in the system of UBE2D3/EB complex (Figure 6D), further implying that EB and UBE2D3 formed strong interactions.

Molecular docking simulations visualized the binding state of EB and UBE2D3. From the proposed binding model, it can be found that the stereoconformation of EB fits well with the active Cys85 residue as binding site (Figure 6E), and that the hydroxyl and carbonyl groups in EB forms hydrogen bonds with Ile84 and Leu89 residues of UBE2D3, respectively (Figure 6F). It was reported that Cys85 residue accepts activated Ub from E1 as an E2 active site and conserved in UBE2D3 in different species including humans, mice, rats, and rhesus monkey (Figure S3C). As a sesquiterpene lactone compound, EB contains α,β‐unsaturated carbonyl parts that can covalently binds to free cysteine residues of UBE2D3 at Cys85 by Michael addition. Considering that EB can bind to UBE2D3 and inhibit IκBα degradation and NF‐κB activation based on the finding above, we conclude that a covalent adduct of EB with Cys85 was formed via Michael addition, thereby inactivating UBE2D3.

### 2.7 Silencing of *Ube2d3* impairs the EB mediated anti‐inflammatory protective effect in activated RAW264.7 cells

**FIGURE 7:**
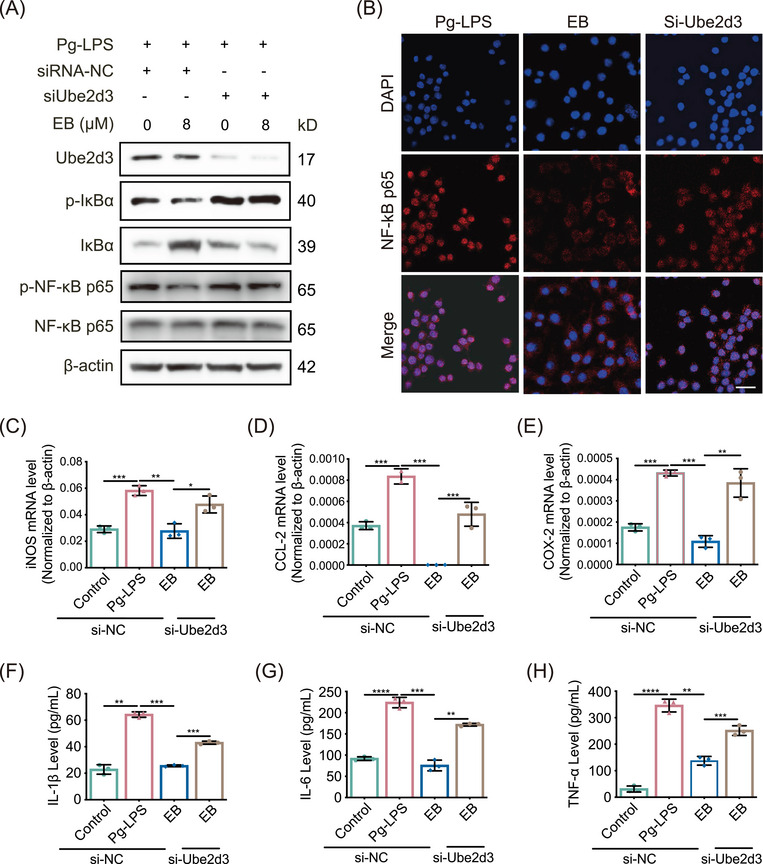
Knockdown of Ube2d3 inhibits the anti‐inflammatory activity of Eupalinolide B (EB). (A) The indicated proteins of Raw264.7 cells transfected with si‐NC or si‐Ube2d3 were detected using western blot after EB treatment under activation with *Porphyromonas gingivalis*‐lipopolysaccharide (Pg‐LPS) for 24 h (*n* = 3). (B) The distribution of nuclear transcription factor‐κB (NF‐κB) p65 were detected using immunofluorescence staining after treatment with EB (*n* = 3). Scale bar: 50 µm. (C‒E) The relative mRNA levels of indicated genes were measured using qRT‐PCR (*n* = 3). (F‒H) The indicated cytokines released in cell culture supernatant were measured by enzyme‐linked immunosorbent assay (*n* = 3). Data are represented as means ± SEM. ^*^ *p* < 0.05, ^**^ *p* < 0.01, ^***^ *p* < 0.001, or ^****^ *p* < 0.0001 was significant; ns, not significant.

**FIGURE 8:**
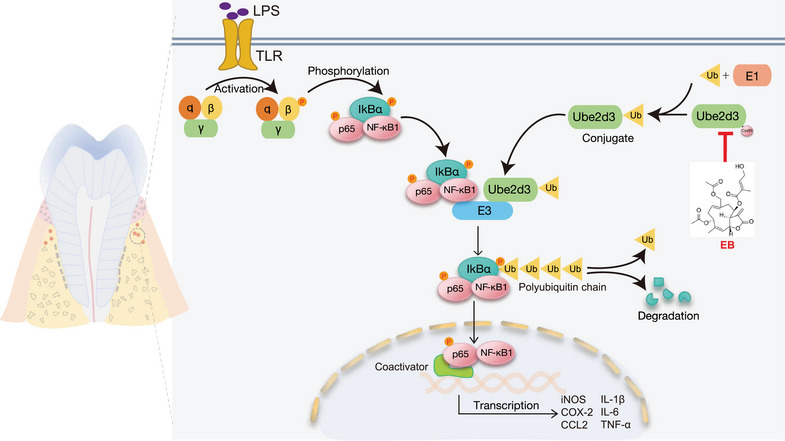
The molecular mechanism proposed for the anti‐periodontal inflammation activity of Eupalinolide B (EB).

To further investigate the specific role of UBE2D3 underlying the anti‐inflammatory activity and effect of EB on periodontitis, we transfected siRNA in Raw264.7 cells to knockdown (KD) *Ube2d3*. Compared with the si‐NC control group, si‐*Ube2d3* significantly inhibited UBE2D3 expression in protein level (Figure 7A). After exposure to EB, phosphorylated IκBα was decreased under Pg‐LPS stimulation while substantially upregulated after *Ube2d3* KD. However, it was observed that IκBα significantly decreased in protein level in the *Ube2d3* silenced group (Figure 7A). The effect of EB on the activation of NF‐κB was further investigated by WB and the results displayed that EB effectively reduced the level of activated NF‐κB p65 (p‐NF‐κB p65) induced by Pg‐LPS in activated Raw264.7 cells. However, in the *Ube2d3* silencing group, the inhibitory effect of EB on NF‐κB activation was significantly reduced (Figure 7A). Besides, EB significantly inhibited the nuclear translocation of NF‐κB p65 by immunofluorescence staining under stimulation by Pg‐LPS while *Ube2d3* KD reduced the inhibitory effects (Figure 7B). The experiments above showed that *Ube2d3* KD specifically blocked dephosphorylation and stabilization of IκBα induced by EB treatment, thereby reversing the inhibitory effect of EB on the phosphorylation and stabilization of NF‐κB p65. To examine the consequence of increased NF‐κB‐driven transcriptional activity in *Ube2d3* silenced Raw264.7 cells, the real‐time qPCR was used to detect levels of genes participated in NF‐κB signaling pathway. And result showed that *Ube2d3* KD significantly reversed the downregulation of the downstream genes of NF‐κB such as *iNOS*, *CCL‐2*, and *COX‐2* (Figure 7C‒E). Furthermore, ELISA was used to determine the secretion levels of cytokines IL‐1β, IL‐6, and TNF‐α, downstream of the NF‐κB pathway (Figure 7F‒H). Consistently, compared to the control, the repression of cytokines release by EB treatment was also reversed in *Ube2d3* silenced group. The results above displayed that the inhibitory effect of EB on cellular inflammation was mediated by UBE2D3 protein. In the present study, we demonstrated that EB covalently binds to Cys85 residue of UBE2D3, effectively inhibits the activity of UBE2D3 and prevents the degradation of IκBα and the activation of NF‐κB, inhibiting the expression of downstream inflammatory genes and the release of pro‐inflammatory cytokines and finally inhibiting the progression of periodontal inflammation (Figure 8). In general, our study provide a promising drug candidate to treat periodontitis.

## 3 DISCUSSION

Periodontitis is kind of common oral disease worldwide characterized by irreversible loss of alveolar bone and persistent inflammation in periodontal tissue.^47^, ^48^ Oral bacteria and the dysregulation of host immune‐inflammatory response might further induced several other chronic systemic diseases.^49^, ^50^, ^51^, ^52^ In view of the harm of periodontitis, it is important to study the prevention and treatment for periodontitis. Clinical therapy for periodontitis contains non‐surgical treatment, surgical treatment, and adjunctive drug therapy. With the aim of improving the outcome of non‐surgical or surgical treatment, the drug therapy including systemic/local antibiotics, non‐steroidal anti‐inflammatory drugs, and anti‐cytokine regents were used as adjunctive approaches.^9^, ^53^, ^54^ However, owing to persistent inflammation and activated host immune response in periodontitis, the benefits of adjuncts to conventional periodontal therapy do not achieve the desired efficacy and remaining controversial. A high efficient therapy should satisfy the needs that it can ameliorate local inflammation, alleviate alveolar bone destruction, and regulate the responses of the innate immune system. In the last years, the use of natural agents with properties of anti‐inflammatory, anti‐microbial, and anti‐oxidant from herbals medicine have gain special attention as therapeutical strategy against periodontitis.^55^, ^56^, ^57^, ^58^

EB is considered as an promising therapeutic agent for treatment of inflammatory diseases and cancer.^41^, ^42^, ^43^ Here, for the first time, we explore the protective role of EB in inhibiting periodontal inflammation and alleviating alveolar bone loss in experimental periodontitis samples from C57BL/6 mice as well as its underlying mechanisms. We have synthesized EB‐P to identify potential target proteins of EB. We found that EB directly binds to cys85 residues of UBE2D3 to inhibit its activity in mediating IκBα ubiquitination degradation, stabilizing IκBα and then inhibit the activation of NF‐κB pathway and the expression of downstream genes and cytokines, thus inhibiting inflammation. Silencing *Ube2d3* in activated RAW264.7 cells mitigated the inhibition of EB in NF‐κB pathway activation (Figure 7). Our findings suggest that EB improves inflammation and bone destruction in periodontitis by directly targeting UBE2D3. Therefore, our findings suggest that EB is a novel drug for the treatment of periodontitis.

It has been reported in recent studies that UBE2D3 participated in the regulation of different inflammatory diseases and cancer, making it an attractive molecular drug target.^37^, ^38^, ^59^, ^60^, ^61^ Our data not only found that EB directly binds to UBE2D3, but also identified the amino acid sites that specifically bind to UBE2D3. Unlabeled molecular interaction experiments based on unlabeled BLI technology showed EB can bind to UBE2D3 with a dissociation constant of 9.25 µM. Combined with high‐resolution mass spectrometry, we found that EB directly binds to the Cys85 residue of UBE2D3, which is essential for its ubiquitin binding activity. Molecular docking and molecular dynamics simulation assays further demonstrated that the α‐β‐unsaturated alkenyl group of EB can form a covalent adduct with Cys85 through Michael addition reaction, finally inactivating UBE2D3 and inhibiting the ubiquitination and degradation of IκBα and activation of NF‐κB. Therefore, our study shows that EB is an effective compound as the adjunctive therapy for periodontitis treatment. In addition, combination therapy using different classes of drugs may be a promising candidate for periodontal treatment.

Nevertheless, we must interpret the results above with caution and some limitations should be taken into account. First, the inhibition specificity of EB for UBE2D3 over other ubiquitin coupled enzymes (E2s) remain further exploration. Second, in vitro ubiquitination reaction needs to be conducted to directly test EB's activity against the formation of polyUb chains. Third, in addition to NF‐κB signaling pathway, UBE2D3 is reported to be involved in a number of inflammatory signaling pathways, including RIG‐I signaling pathway^62^, ^63^ P53 and DNA repair signaling pathway.^35^, ^64^, ^65^ Therefore, whether EB inhibits periodontitis through these pathways remains to be further elaborated. Fourth, regarding EB's therapeutic potential, significant issues remain, including that EB's insolubility and poor bioavailability limited its clinical application. Fifth, the association between UBE2D3 and periodontitis requires collection of human samples from clinical healthy groups and periodontitis groups. And more well‐designed clinical studies are needed to provide evidence on the association between UBE2D3 and periodontitis. Sixth, further studies are needed to explore whether long‐term systemic application of EB can cause toxic and side effects, and we are currently conducting relevant studies on local administration of EB in specific dosage forms for treatment of periodontitis. Besides, EB can also inhibit osteoclast production, so there may be other molecular targets related to osteoclast differentiation, which is also the focus of future research.

## 4 MATERIALS AND METHODS

### 4.1 Cells

The Raw264.7 cells were purchased from the Chinese Academy of Medical Sciences and cultured in high glucose Dulbecco's minimum essential medium (DMEM) supplemented with 10% fetal bovine serum at 37°C with 5% CO_2_. The cells were identified using short tandem repeat markers from Fuheng Bio (last tested in 2023). For the experiments of inducing inflammation, Raw264.7 cell was stimulated with 10 µg/mL Pg‐LPS (InvivoGen) based on the preliminary experiment (data not shown) and exposed to EB (PUSH Bio) or EB‐P for 0–24 h.

### 4.2 Mice experimental periodontitis models

The mice (male, 8‐week old) experimental periodontitis model were established on C527BL/6 background and the mice were divided into three groups randomly as blank control group, ligated periodontitis model control group, and EB administration group. After one week of adaptive feeding, silk ligations were performed on the maxillary second molar teeth to establish an experimental periodontitis model. The ligated mice were then intraperitonelly injected with EB (30 mg/kg) or equivalent normal saline every day for 14 days based on previous research and data^41^ and the silk was removed on the eighth day. Standard food and water were freely available to all mice and the animal experiments were approved by the Institutional Animal Care and Use Committee (IACUC) of Shenzhen People's Hospital with a certificate number of AUP‐230725‐KWH‐0286‐01.

### 4.3 Quantitative proteome analysis

Gingival tissues adjacent to the second molar are removed and lysed in 8 M urea/50 mM TEAB in order to extract protein lysate. Then, the bicinchoninic acid (BCA) kit was used to measure the protein concentration. Next, dithiothreitol (DTT) and IAA were add and incubated for 30 min separately. Then, proteins were digested by trypsin overnight and conducted for dimethylation labeling. More specifically, peptides were redissolved in 100µL of TEAB, and 8 µL of 4% D13CDO (heavy label group), DCDO (medium label group), or HCHO (light label group) was added to different groups of samples. Subsequently, 8 µL of 0.6 M NaBH3CN (winning and light group) and 8 µL of 0.6 M NaBD3CN (heavy group) were added on ice and the reaction was performed for 1.5 h at 25°C. After terminating the reaction, samples with the same volume were combined and desalted with a commercial C18 column and analyzed using orbitrap fusion lumos (Thermo Scientific).^66^

### 4.4 Radiographic analysis (micro‐computed tomography)

Mouse alveolar bone tissue specimens were fixed in 4% paraformaldehyde solution. The microstructure of the mouse maxilla were scanned using micro‐computed tomography (SkyScan 1176) with a resolution of 9 µm. CTVOX software was used for 3D reconstruction. Scanning parameters of bone tissue in the target region including the trabecular bone volume/tissue volume ratio (BV/TV) and the CEJ‒ABC were measured using CTAn software. The analytical range of all specimens remained consistent.

### 4.5 Histological analyses

Alveolar bone tissue specimens were decalcified using 10% EDTA solution. After a dehydration by automatic tissue dehydrator, the alveolar bone specimens were then embedded using paraffin and subsequently sliced to sections with 4 µm thickness. The sections were then subjected to TRAP staining and HE staining and examined and photographed by microscope, and the quantitation of TRAP‐positive osteoclasts were analyzed using ImageJ software.

### 4.6 Detection of cytokines by ELISA

To measure the level of cytokines of IL‐6, TNF‐α, and IL‐1β in RAW 264.7 cells released in cell culture medium, the cells were incubated with EB or EB‐P probe (4 or 8 µM) for 24 h after stimulated by Pg‐LPS (10 µg/mL). The supernatant of medium were collected, assessing using ELISA kits as the manufacturer's protocol described (Abclonal Biotech).

### 4.7 Cell viability test

Cells cultured in DMEM medium were treated with EB at concentrations of 0–32 µM for 24 h, and the cell viability was examined normalized with the control group by CCK‐8 method.

### 4.8 Immunoblotting

The cells were treated with Pg‐LPS and EB as described above, and homogenized with an ultrasonic crusher. After centrifugation at high speed, the supernatant was collected and quantified by BCA method. An amount of 50 µg protein solution was separated by SDS‐PAGE electrophoresis, transferred to the polyvinylidene fluoride (PVDF) membrane, blocked in skim milk and incubated with anti‐UBE2D3 antibody (1:1000, Cell Signaling Technology, 4330S), anti‐p‐NF‐κB p65 antibody (1:1000, Cell Signaling Technology, 3033), anti‐NF‐κB p65 antibody (1:1000, Proteintech, 80979‐1‐RR), anti‐p‐IκBα antibody (1:1000, Cell Signaling Technology, 2859), anti‐IκBα antibody (1:1000, Cell Signaling Technology, 4814), or anti‐β‐actin antibody (1:5000, Proteintech, 81115‐1‐RR) overnight at 4°C. Finally, target protein bands were detected by enhanced chemiluminescent assay after incubation with appropriate secondary antibody.

### 4.9 Real‐time quantitative PCR

Total RNA of tissues and Raw264.7 cells were extracted using Trizol universal reagent (Tiangen, DP405). Reverse transcription and SYBR green qPCR were conducted as manufacturer's instructions (Transgen, AQ601) with a real‐time fluorescence qPCR instrument (ThermoFisher, Quant Studio 5). All primer sequences used here are demonstrated in Table S4.

### 4.10 Immunofluorescence staining

For co‐localization imaging of target protein and EB‐P, Raw264.7 cells were activated with Pg‐LPS and incubated with or without EB‐P (8 µM) for 4 h as described above. Cells were fixed by 4% paraformaldehyde and permeabilized by 0.2% Triton X‐100. After blocked using 5% BSA, incubated with anti‐UBE2D3 antibodies and secondary antibodies, pre‐click reaction mix was added into cells for click reactions.^67^ For imaging of EB‐P, Raw264.7 cells were stimulated and click chemical reactions were performed as described above, then cells were washed by Tris‐Borate‐Sodium Tween‐20 buffer and stained by 4', 6‐diamidino‐2‐phenylindole and scanned with confocal microscopy (Leica TCS SP8 SR).

### 4.11 Labeling and competition experiments

For in vitro fluorescence labeling and competition experiments in cell protein lysates, activated Raw264.7 cell precipitation was collected and lysed using 0.1% Triton/PBS with protease inhibitors cocktail. The groups are as follows: blank control group, EB‐P probe labeling group, and EB pre‐treatment competition group. The competition group was pretreated with 400 µM EB for 1 h, and then 0 µM probe, 50 µM probe, and 50 µM probe were, respectively, added to the three groups and incubated at 37°C for labeling reaction. Next, click reaction mix were added for reaction at 29°C as described above. The protein was precipitated by cold acetone method and separated by SDS‐PAGE electrophoresis, followed by fluorescence imaging and Coomassie bright blue staining.

For in vivo labeling and competition experiments, 8‐week C57BL/6 male mice were randomly divided as control group (equivalent DMSO), EB‐P labeling group (0.5 mg/kg EB‐P), and EB competition groups (2.5, 5, and 10 mg/kg/day EB with 0.5 mg/kg EB‐P). Then, EB (2.5, 5, and 10 mg/kg/day, 1 µL each) was pre‐injected into the gingival tissue at interdental site between the first and second molar of the maxilla, and the control group was injected with DMSO (1 µL) at the same site simultaneously for 6 h. Then, 0.5 mg/kg EB‐P was subsequently injected into the gingival tissues for 6 h following EB pre‐injection. The gingival tissues were separated and homogenized in 0.1% Triton/phosphate buffer solution (PBS) with protease inhibitors cocktail to extract total protein and normalized by β‐actin using WB. Subsequently, the click reaction mix containing Biotin‐N3 were added to the protein solution for reaction at 29°C for 1 h as described above. Then, protein were precipitated using cold acetone method and redissolved with 0.1% SDS. The protein supernatant was then incubated with streptavidin agarose at 4°C overnight to pull down the protein‒probe complex and separated by SDS‐PAGE electrophoresis, followed by WB using anti‐UBE2D3 antibody.

### 4.12 Protein targets identification

To identify the target proteins of EB, we conducted a pull‐down experiment followed by liquid chromatography‐mass spectrometry/mass spectrometry analysis. RAW264.7 cells were pre‐incubated with EB and incubated with EB‐P, with (competetion group) or without EB (probe labeling group). Then, the click reaction mix containing Biotin‐N3 were added to the protein solution. After a reaction for 1 h, the protein was precipitated and redissolved with 0.1% SDS. After centrifugation at high speed, the protein supernatant was then incubated with streptavidin beads at 4°C overnight. Then, protein‒bead complex were collected and digested to peptides for dimethylation labeling.^66^, ^67^ Finally, the identification was performed with the Orbitrap Fusion Fluorescence Spectrometer (Thermo Scientific). The differentially expressed proteins between the competition group and the probe group were selected for pathway and visualization analysis. For a pull‐down WB experiment, the pull‐down procedures were performed as above. Then, protein were redissolved by protein loading buffer for WB assay.

### 4.13 Plasmids and protein purification

Wild type UBE2D3 (NM_025356.5) and mutated UBE2D3 (Cys85 to Ala85) were inserted into pET30a vector and transformed into BL21(DE3) expressing strain. The expression of UBE2D3 protein was induced by adding 0.2 mM IPTG. The bacteria were ultrasonically lysed. The protein was purified through Ni‐IDA affinity chromatography and the target protein was eluted with imidazole‐containing solution and collected for SDS‐PAGE analysis. The protein was collected and dialyzed, then the supernatant was filtered by a 0.22 um filter. The purity of recombinant protein above 90% was analyzed with CBB staining.

### 4.14 RNA interference assay

Sequences of *Ube2d3* and negative control siRNAs (Table S5) were synthesized by Raybio. Cells were cultured and transfected following manufacturer's instructions. Next, cells were stimulated by Pg‐LPS and treated with EB for 24 h as previously described and protein or cell culture supernatant were extracted for further determination by WB or ELISA.

### 4.15 Molecular docking simulation

The crystal structure of UBE2D3 (PDB: 5eGG) as PDB format was downloaded from Protein Data Bank (PDB, https://www.rcsb.org). Then, the binding mode of EB‐UBE2D3 complex was optimized, the UBE2D3 protein and EB were parameterized using the OPLS4 force field. After preprocessing of the EB‐UBE2D3 complex as described, an unrestricted simulation was performed for 100 ns. The interactions were analyzed and dynamic trajectory animations were generated using Maestro 2023 (Schrödinger, version 9.0).

### 4.16 Cellular thermal shift assays

Raw264.7 cells were divided into control group (dimethyl sulfoxide) and 400 µM EB treatment group (EB). After co‐incubation of the protein lysate and drug, each group was divided into multiple microtubules and heated in a PCR apparatus, and the gradient was set at 42°C‒87°C. The samples in tubes were subsequently centrifuged (20,000 g), then supernatant were subjected to WB assay using UBE2D3 antibody as described above.

### 4.17 Identification of binding site of UBE2D3 to EB

To determine the specific binding sites of EB to UBE2D3, 30 µg recombinant UBE2D3 in PBS was incubated with or without 1 mM EB at 37°C for 4 h. Then to reduce and alkylate proteins, 10 mM DTT and 20 mM IAA were separately added and incubated at 37°C for 30 min protected from light. The proteins were then digested into peptides with mass spectrometric trypsin following the manufacturer's instructions. The peptides were analyzed by liquid chromatography coupled with mass spectrometry‐based methods after desalted with C18 column. The data and the peptide sequences were analyzed using the pFind tool.^46^

### 4.18 Measurement of the affinity between UBE2D3 and EB

The interactions between EB and UBE2D3 was measured with Octet R2 (Sartorius) based on a BLI method. The PBS was used as the assay buffer and the experiment was performed at 30°C. To reduce non‐specific interactions, 0.1% BSA and 0.02% Tween 20 were added in the assay. And to increase compound solubility, 2% dimethyl sulfoxide was added to reaction mix. Purified UBE2D3 was bond onto NTA Biosensors (Sartorius). The binding assays were conducted at concentrations of EB from 0.8 to 200 µM. The Octet Red software provided by the manufacturer was used to determine the binding constants based on global fits of the binding curves. A 1:1 model was chosen to analyze the data and calculate the *K*_d_ values.

### 4.19 Statistical analysis

Statistical analysis of data was performed by Student's *t*‐test between groups or by one‐way ANOVA followed by the Tukey's test in multiple groups. All experiments were performed for at least three independent biological replicates. Statistical analysis was conducted using GraphPad Prism 9.0 software. Data are represented as means ± standard error of meam. ^*^ *p* < 0.05, ^**^ *p* < 0.01, ^***^ *p* < 0.001, or ^****^ *p* < 0.0001 was significant; ns, not significant.

## AUTHOR CONTRIBUTIONS

W.K. and R.Z. conceived the project, conducted the experiments, and wrote the manuscript. P.S. and L.Y. conceived the project and revised the manuscript. S.Z. performed the bioinformatics analysis and interpreted data. Y.Z. designed and synthesized the EB‐probe. J.Z. performed MS experiments. R.C., Y.W., and D.L. performed the mouse model construction, cell culture, and ELISA experiments. Y.K.W., Z.G., and P.W. conceived the in vivo experiment and revised the manuscript. X.O. and J.W. supervised experiments, conceptualized the project, and revised the manuscript. All authors have read and approved the final manuscript.

## CONFLICT OF INTEREST STATEMENT

The authors declare they have no conflicts of interest.

## ETHICS STATEMENT

All the in vivo experiments were approved and supervised by the Animal Ethics Committee of Shenzhen People's Hospital (approval ID: AUP‐230725‐KWH‐0164‐01).

## Supporting information



Supporting Information

Supporting Information

## Data Availability

All data relevant to the study are included in the article or uploaded as Supporting Information.
