# Supplementary material for: Eupalinolide B inhibits periodontitis development by targeting ubiquitin conjugating enzyme UBE2D3
Source: MedComm (2020). 2025 Jan 14;6(1):e70034. doi: 10.1002/mco2.70034 (PMC11731104; doi:10.1002/mco2.70034)
Supplement: Supplementary file 1 — Supporting Information [file MCO2-6-e70034-s001.docx]

**Eupalinolide B inhibits periodontitis development by targeting ubiquitin conjugating enzyme UBE2D3**

(**Short running title**: Anti-periodontitis role of Eupalinolide B targeting UBE2D3)

Wenhua Kuang ^a, #^, Ruishen Zhuge ^b, #^, Ping Song ^c, #^ , Letai Yi ^d,^ ^#^, Shujie Zhang ^e,^ , Ying Zhang ^e^ , Yin Kwan Wong ^a^，Ruixing Chen ^f^, Junzhe Zhang ^e^ , Yuanbo Wang ^b^ , Dandan Liu ^e^ , Zipeng Gong ^f,^ * Peili Wang ^c,^ * , Xiangying Ouyang ^b,^ * and Jigang Wang ^a, e, g,^ *

a. Department of Urology, Shenzhen Clinical Research Centre for Geriatrics, Shenzhen People's Hospital; The First Affiliated Hospital, Southern University of Science and Technology, Shenzhen 518020, Guangdong, China.

b. Department of Periodontology, Peking University School and Hospital of Stomatology, National Clinical Research Center for Oral Diseases, National Engineering Laboratory for Digital and Material Technology of Stomatology, Beijing Key Laboratory of Digital Stomatology, Beijing, China.

c. National Clinical Research Center for Chinese Medicine Cardiology, Xiyuan Hospital, China Academy of Chinese Medical Sciences, Beijing, China

d. Inner Mongolia Medical University, Hohhot 010000, Inner Mongolia, China.

e. State Key Laboratory for Quality Ensurance and Sustainable Use of Dao-di Herbs, Artemisinin Research Center, and Institute of Chinese Materia Medica, China Academy of Chinese Medical Sciences, Beijing 100700, China.

f. State Key Laboratory of Functions and Applications of Medicinal Plants, Guizhou Provincial Key Laboratory of Pharmaceutics, Guizhou Medical University, Road, Guiyang, 550004, China

g. State Key Laboratory of Antiviral Drugs, School of Pharmacy, Henan University, Kaifeng 475004, China

*Correspondence: wangjigang@u.nus.edu; [kqouyangxy@bjmu.edu.cn](mailto:kqouyangxy@bjmu.edu.cn); [qiexuxing0721@163.com;](mailto:191593690@qq.com;) gzp4012607@126.com.

# Wenhua Kuang, Ruishen Zhuge, Ping Song and Letai Yi contributed equally to this work.

**
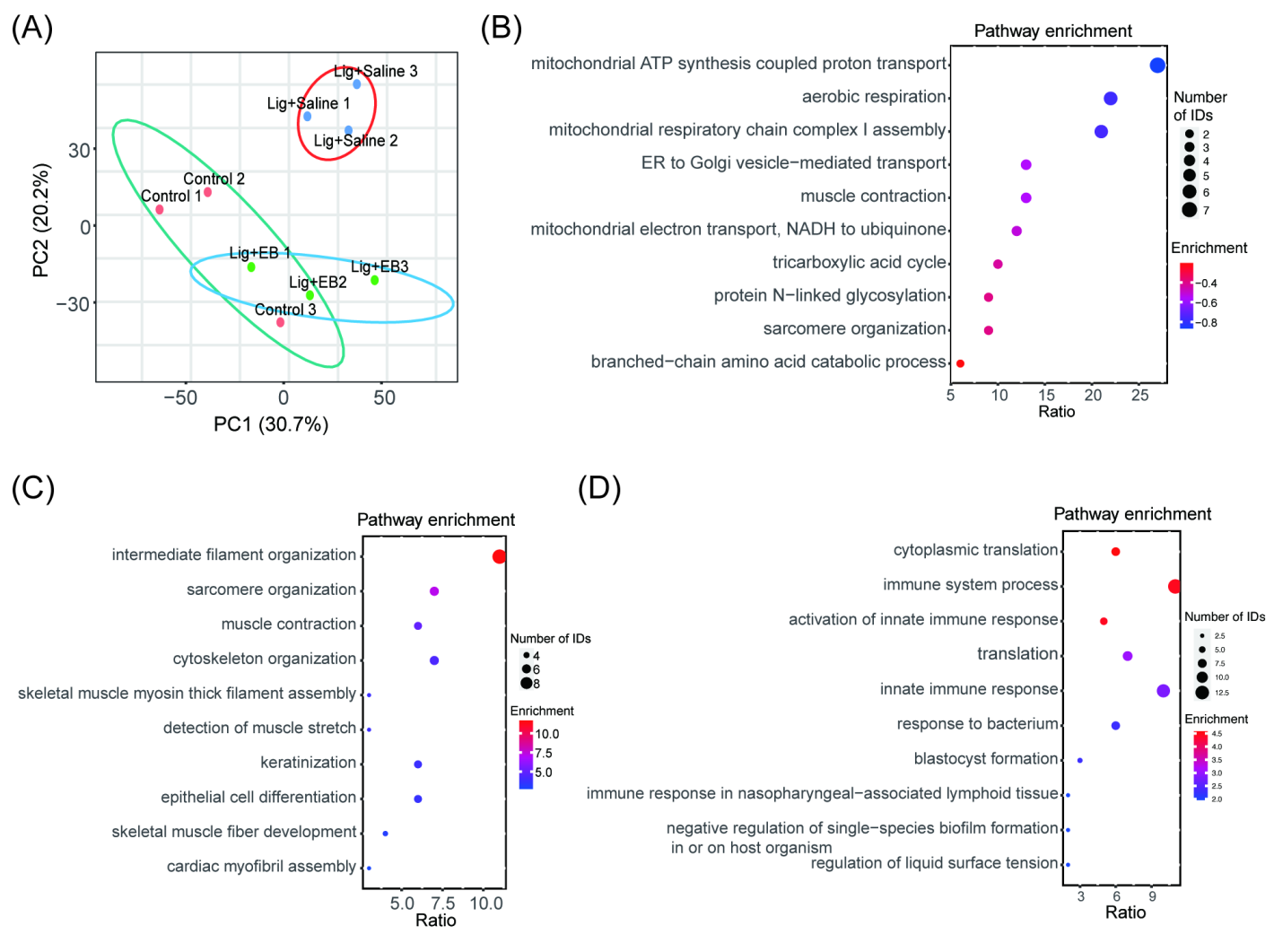
FIGURE S1 Proteome in mouse gingival tissues identifies significantly-regulated pathways.** (A) PCA of proteome in ligated periodontal groups with or without EB treatment and normal control groups represented in a two-dimensional space, n=3. (B) Gene ontology analysis for the significantly up-regulated pathways by EB in gingival tissues compared to model group. The top ten regulated pathways were demonstrated. (C-D) Gene ontology analysis for the significantly down-regulated (C) and up-regulated (D) pathways in periodontitis gingival samples compared to control group. The top ten regulated pathways were demonstrated.

**
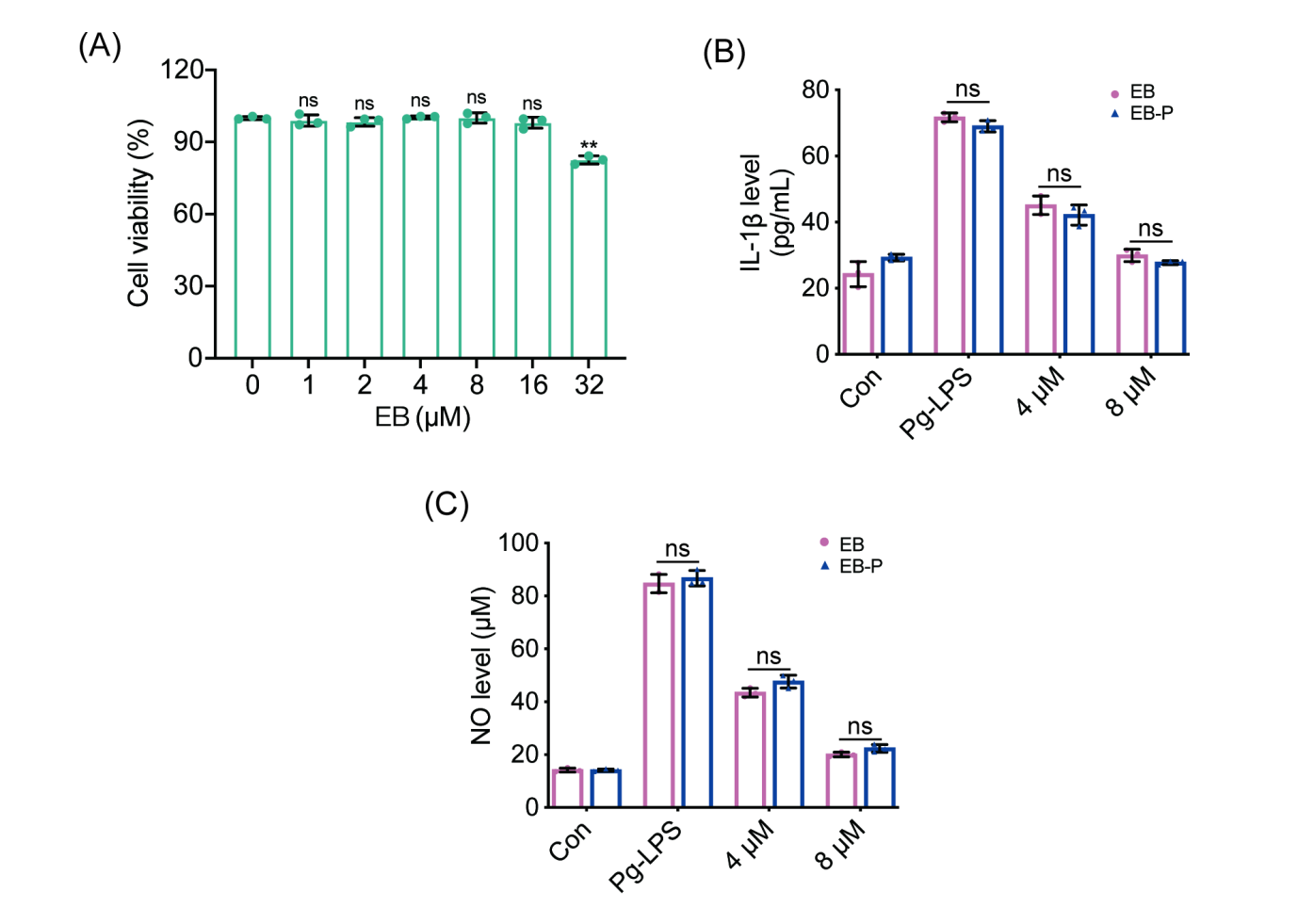
**

**FIGURE S2 The effects of EB on cell viability and the bioequivalence of EB and EB-P in anti-inflammatory role in vitro.** (A) Cell viability of Raw264.7 cells treated with EB, n=3. (B) Release of inflammatory cytokine IL-1 β in Pg-LPS-induced RAW264.7 cells. (C) Release of NO in medium supernatant of Pg-LPS-induced RAW264.7 cells.


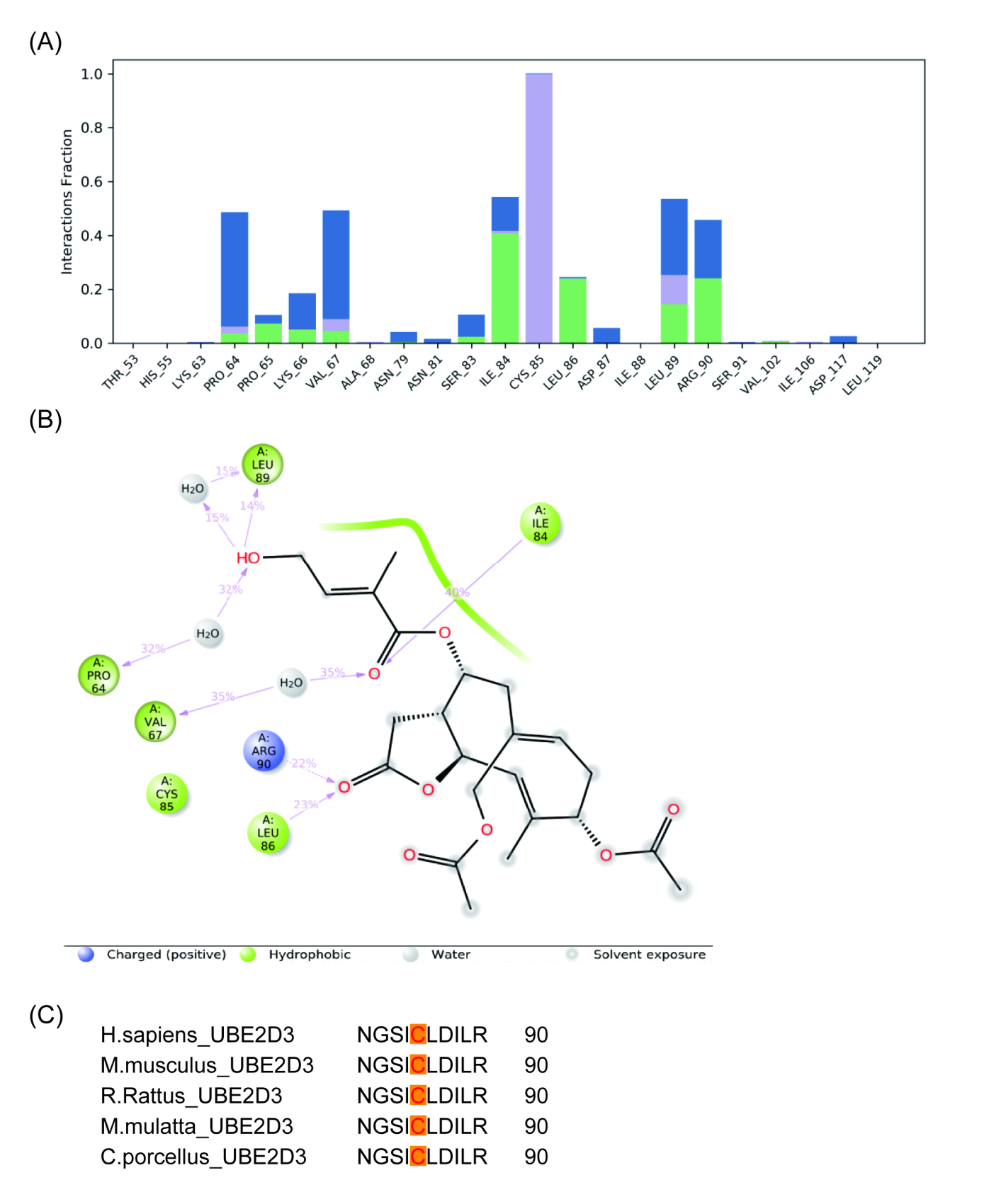


**FIGURE S3 Molecular interactions of EB with UBE2D3 generated from the last frame of the MD simulation.** (A-B) Interaction fraction of the UBE2D3/EB complex. (C) Multiple sequence alignment showed that Cys85 position (Red) is highly conserved in different species of UBE2D3.

**Table S2 Found peptides (without EB modification).**

| Found peptides (without EB modification) | | | | |
| --- | --- | --- | --- | --- |
| Exp.MH+ | Calc.MH+ | Sequence | Modification | Miss.Clv.Sites |
| 2099.039947 | 2099.070138 | IYHPNINSNGSICLDILR | 13,Carbamidomethyl[C]; | 0 |
| 989.544997 | 989.544782 | SICLDILR | 3,Carbamidomethyl[C]; | 0 |
| 1046.567773 | 1046.566243 | GSICLDILR | 4,Carbamidomethyl[C]; | 0 |
| 2099.073742 | 2099.070138 | IYHPNINSNGSICLDILR | 13,Carbamidomethyl[C]; | 0 |
| 2099.072058 | 2099.070138 | IYHPNINSNGSICLDILR | 13,Carbamidomethyl[C]; | 0 |
| 2099.07253 | 2099.070138 | IYHPNINSNGSICLDILR | 13,Carbamidomethyl[C]; | 0 |
| 2099.072634 | 2099.070138 | IYHPNINSNGSICLDILR | 13,Carbamidomethyl[C]; | 0 |
| Mached peptides in UBE2D3 shown in red: MALKRINKELSDLARDPPAQCSAGPVGDDMFHWQATIMGPNDSPYQGGVFFLTIHFPTDYPFKPPKVAFTTRIYHPNINSNGSICLDILRSQWSPALTISKVLLSICSLLCDPNPDDPLVPEIARIYKTDRDKYNRISREWTQKYAM | | | | |
|  |  |  |  |  |
|  |  |  |  |  |
|  |  |  |  |  |

**Table S3 Found peptides (with EB modification).**

| Found peptides (with EB modification) | | | | |
| --- | --- | --- | --- | --- |
| Exp.MH+ | Calc.MH+ | Sequence | Modification | Miss.Clv.Sites |
| 2504.241635 | 2504.237639 | IYHPNINSNGSICLDILR | 13,eB; | 0 |
| 1565.779438 | 1565.776666 | NGSICLDILR | 5,eB; | 0 |
| 1394.716119 | 1394.712283 | SICLDILR | 3,eB; | 0 |
| 1451.737252 | 1451.733744 | GSICLDILR | 4,eB; | 0 |
| 1879.941205 | 1879.935671 | INSNGSICLDILR | 8,eB; | 0 |
| 2504.24055 | 2504.237639 | IYHPNINSNGSICLDILR | 13,eB; | 0 |
| 2504.240941 | 2504.237639 | IYHPNINSNGSICLDILR | 13,eB; | 0 |
| 2504.241073 | 2504.237639 | IYHPNINSNGSICLDILR | 13,eB; | 0 |
| 2504.242209 | 2504.237639 | IYHPNINSNGSICLDILR | 13,eB; | 0 |
| 1652.813118 | 1652.808691 | SNGSICLDILR | 6,eB; | 0 |
| Mached peptides in UBE2D3 shown in red: MALKRINKELSDLARDPPAQCSAGPVGDDMFHWQATIMGPNDSPYQGGVFFLTIHFPTDYPFKPPKVAFTTRIYHPNINSNGSICLDILRSQWSPALTISKVLLSICSLLCDPNPDDPLVPEIARIYKTDRDKYNRISREWTQKYAM | | | | |
|  |  |  |  |  |
|  |  |  |  |  |
|  |  |  |  |  |

**Table S4 Sequences of primers for q-PCR.**

| Genes | Forward primer (5’-3’) | Reverse primer (5’-3’) |
| --- | --- | --- |
| TNF-a | CCACACGCTCTTCTGTCTA | GCCATGGAACTGATGAGAGG |
| IL-1β | CAGGATGAGGACATGAGCACC | CTCTGCAGACTCAAACTCCAC |
| IL-6 | GCTACCAAACTGGATATAATCAGGA | CCAGGTAGCTATGGTACTCCAGAA |
| iNOS | CCCTTCCGAAGTTTCTGGCAGCAG | GGCTGTCAGAGCCTCGTGGCTTTGG |
| COX-2 | TCACCTGCTGCTACTCATTC | TACAGAAGTGCTTGAGGTGG |
| CCL-2 | AACTGCATCTGCCCTAAGGT | AGGCATCACAGTCCGAGTCA |

**Table S5 Sequences of primers for siRNA.**

| Gene | Sense (5’-3’) | Anti-sense (3’-5’) |
| --- | --- | --- |
| Si-Ube2d3 | CAGAGAUAAGUACAACAGAAU | AUUCUGUUGUACUUAUCUCUG |
| Si-NC | UUCUCCGAACGUGUCACGU | ACGUGACACGUUCGGAGAA |
